# Needle tract seeding in renal tumor biopsies: experience from a single institution

**DOI:** 10.1186/s13000-021-01106-2

**Published:** 2021-05-16

**Authors:** Yan Zhou, Paari Murugan, Faqian Li, Lihong Bu

**Affiliations:** Department of Laboratory Medicine and Pathology, University of Minnesota, 420 Delaware St. SE, 55455 Minneapolis, MN USA

**Keywords:** Biopsy needle tract, Tumor seeding, Renal cell carcinoma, Papillary renal cell carcinoma, Clear cell renal cell carcinoma

## Abstract

**Background:**

Percutaneous needle biopsy of renal masses has been increasingly utilized to aid the diagnosis and guide management. It is generally considered as a safe procedure. However, tumor seeding along the needle tract, one of the complications, theoretically poses potential risk of tumor spread by seeded malignant cells. Prior studies on the frequency of needle tract seeding in renal tumor biopsies are limited and clinical significance of biopsy-associated tumor seeding remains largely controversial.

**Methods:**

Here we investigated the frequencies of biopsy needle tract tumor seeding at our institution by reviewing the histology of renal cell carcinoma nephrectomy specimens with a prior biopsy within the last seventeen years. Biopsy site changes were recognized as a combination of foreign body reaction, hemosiderin deposition, fibrosis and fat necrosis. The histologic evidence of needle tract tumor seeding was identified as clusters of tumor cells embedded in perinephric tissue spatially associated with the biopsy site. In addition, association between parameters of biopsy techniques and tumor seeding were investigated.

**Results:**

We observed needle tract tumor seeding to perinephric tissue in six out of ninety-eight (6 %) renal cell carcinoma cases including clear cell renal cell carcinoma, papillary renal cell carcinoma, chromophobe, and clear cell papillary renal cell carcinoma. The needle tract tumor seeding was exclusively observed in papillary renal cell carcinomas (6/28, 21 %) that were unifocal, small-sized (≤ 4 cm), confined to the kidney and had type 1 features. No recurrence or metastasis was observed in the papillary renal cell carcinoma cases with tumor seeding or the stage-matched cases without tumor seeding.

**Conclusions:**

Our study demonstrated a higher than reported frequency of needle tract tumor seeding. Effective communication between pathologists and clinicians as well as documentation of tumor seeding is recommended. Further studies with a larger patient cohort and longer follow up to evaluate the impact of needle tract tumor seeding on long term prognosis are needed. This may also help reach a consensus on appropriate pathologic staging of renal cell carcinoma when the only site of perinephric fat invasion is within a biopsy needle tract.

## Introduction

The indications of and demand for percutaneous needle biopsy of renal tumors have been expanding with rapid advances in medical imaging technology and treatment modalities [1]. Historically, renal mass biopsy (RMB) was limited to differentiate renal cell carcinoma (RCC) from other differential diagnoses including benign tumors, metastatic disease, infection or lymphoma. In contrast, nowadays it is increasingly considered for risk stratification of renal cell carcinoma, as well as for guiding treatment strategies. The 2020 National Comprehensive Cancer Network (NCCN) guidelines recommend RMB of small lesions for diagnosis and stratification of active surveillance, cryosurgery and radiofrequency ablation therapy [2]. The American Urological Association (AUA) guideline also emphasizes that a RMB should be performed prior to ablation therapy to provide pathologic diagnosis and guide subsequent surveillance [3]. In addition, RMB is considered highly accurate for the diagnosis of malignancy and histologic determination of RCC subtypes in several systemic analyses [3–6]. Percutaneous needle core biopsy of renal tumors has been generally considered as a safe procedure. Complications other than hematoma are rare. These include tumor seeding along the needle tract, arteriovenous fistula formation, infection and pneumothorax [7–9]. In particular, tumor seeding along the biopsy needle tract is always a safety issue to consider in biopsy procedures or fine needle aspiration of mass lesions in various tissues, as it poses potential risk of iatrogenic local tumor spread by seeded malignant cells and possible subsequent cancer recurrence or dissemination [1]. Historically, the rate of needle tract tumor seeding in renal biopsy was estimated to be as low as 0.01 % by Smith in 1991, and Herts and Baker in 1995 [9, 10]. To date, only a handful of case reports and a small case series have been published [8, 11–16]. However, the frequency and clinical significance of biopsy-associated tumor seeding remains largely controversial due at least in part to lack of systemic review, histological analysis and follow up data in early studies [9, 10, 17]. In recent years, a few studies re-visited the phenomenon of tumor seeding along core needle biopsy tract in renal cell carcinomas and challenged the previously acknowledged rarity of needle tract tumor seeding following renal tumor biopsy. For example, one case series reported a 1.2 % overall incidence of tumor seeding [16]. In a study of more than 20,000 patients with clinical T1a RCC, the upstaging rate was 2.1 % for patients with prior history of RMB, compared with 1.1 % in patients without prior RMB, although there was no histological evidence showing the association of perinephric fat invasion with a prior biopsy site in this study [18].

Considering the potentially significant impact of tumor seeding, we retrospectively assessed the histologic evidence of tumor seeding along the biopsy needle tract in the resection specimens of renal cell carcinomas at our institution and examined the correlation between tumor seeding and histologic subtypes of renal cell carcinoma.

## Materials and methods

Our institution’s pathology database was searched for cases diagnosed as renal cell carcinoma on biopsy with subsequent nephrectomy from January 2003 to April 2020. To identify patients who underwent both biopsy and nephrectomy, all available medical records and pathology reports were reviewed. A total of 116 patients were identified, including 62 with papillary renal cell carcinoma (PRCC), 71 with clear cell renal cell carcinoma (CCRCC), 4 with clear cell papillary renal cell carcinoma (CCPRCC), and 6 with chromophobe renal cell carcinoma (ChRCC). Of these, the nephrectomy slides of 28 PRCC, 63 CCRCC, 3 CCPRCC, and 4 ChRCC cases (98 cases in total) were available for review. All slides were retrospectively reviewed by two of the authors (LB and YZ) to assess for biopsy site changes and needle tract tumor seeding. The histologic evidence of biopsy site changes on resection specimens included a combination of foreign body reaction, hemosiderin deposition, fibrosis, fat necrosis, and presence of absorbable gelatin [19]. Biopsy needle tract tumor seeding was identified as clusters of tumor cells embedded in perinephric tissue spatially associated with the above-described biopsy site. Fisher’s exact test or t-test was used to compare the rates of biopsy site identification, as well as compare the parameters of biopsy techniques between cases with and without tumor seeding.

## Results

### Tumor seeding is exclusively observed in PRCC

Patients’ demographics and essential pathologic features are summarized in Table 1. The average ages of patients at diagnosis were similar among PRCC, CCRCC and CCPRCC. Patients with ChRCC were relatively younger. The proportions of cases in various pathologic stages (without considering the effect of needle tract tumor seeding) were comparable between CCRCC and PRCC, with pathological T1a stage in more than half the cases of PRCCs and CCRCCs.

**Table 1:** Demographics and pathologic features of the 98 renal cell carcinoma cases with prior biopsy

	Papillary renal cell carcinoma	Clear cell renal cell carcinoma	Clear cell papillary renal cell carcinoma	Chromophobe renal cell carcinoma
**Number of cases**	28	63	3	4
**Age (mean ± SD)**	62.5 ± 11.5	61.1 ± 12.0	60.0 ± 5.6	44.5 ± 6.5
**Sex (Male/Female)**	22/6	36/27	2/1	1/3
**Tumor staging**^**a**^				
pT1a	17	35	3	2
pT1b	4	16	0	1
pT2a	2	1	0	1
pT3a	5	10	0	0
pT3b	0	1	0	0
**Nucleolar grade (ISUP)**				
1	1	6	Not applicable	Not applicable
2	11	38		
3	7	18		
4	0	1		
**Lymphovascular invasion**	1	4	0	0
**Lymph node involvement**	1	1	0	0
**Distant metastasis**	0	1	0	0

^a^ The American Joint Committee on Cancer (AJCC) cancer staging, 8th edition for Tumor

**Fig. 1:**
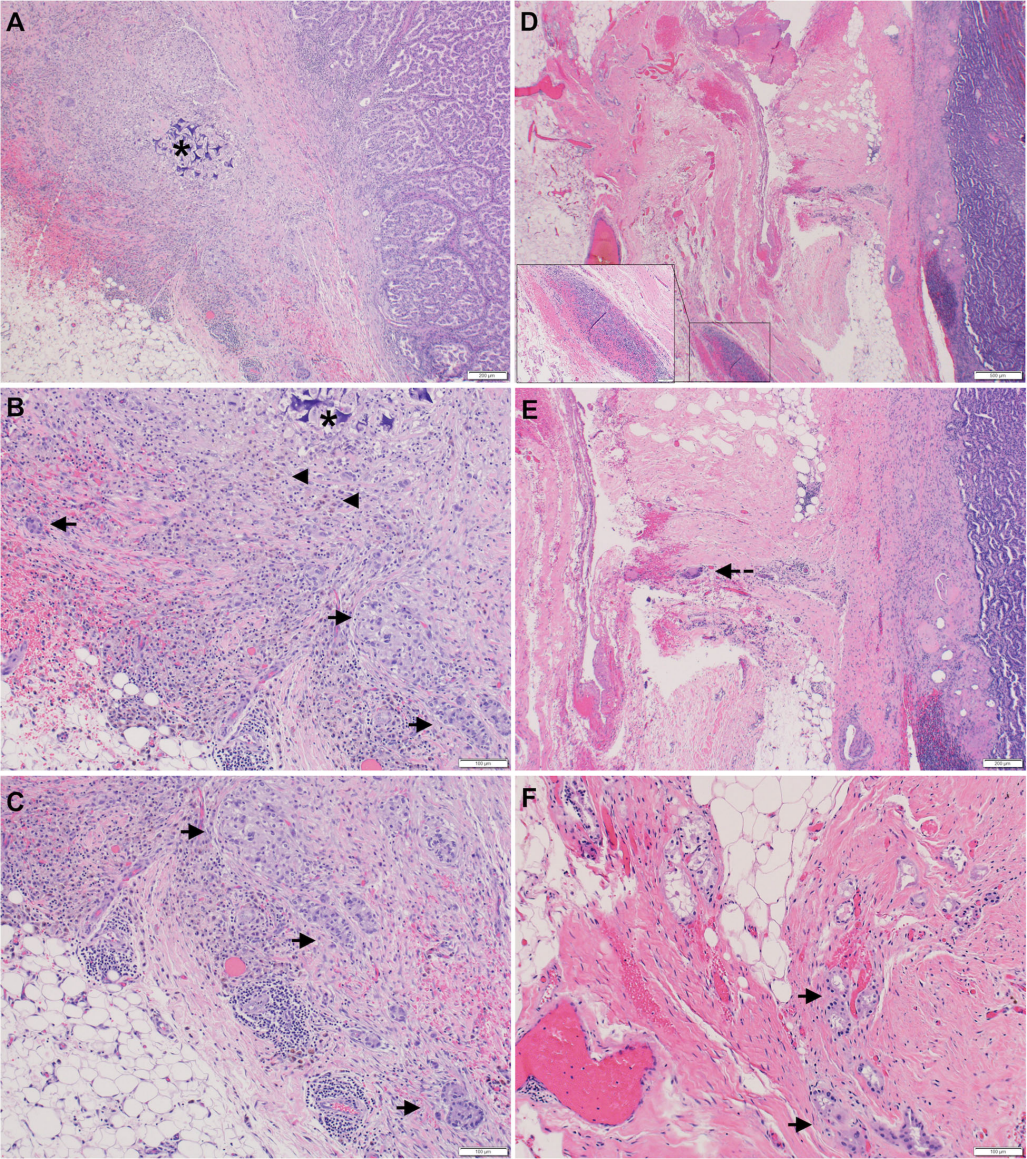
Representative images of two cases (**a**-**c** and **d**-**f**, respectively) demonstrating the histologic evidence of biopsy site changes and tumor seeding along the biopsy site. (**a**) Low power view showing the primary PRCC, perinephric tissue with biopsy site changes and tumor seeding to the adjacent perinephric adipose tissue beyond the renal capsule; (**b**) biopsy site changes showing combination of foreign material deposition (asterisk = gelfoam), foreign body reaction, hemosiderin deposition (arrowhead) and fibrosis, as well as a few clusters of tumor cells (arrow) seeded within the biopsy site; (**c**) High power view highlighting nests of tumor cells (arrow) seeded in the perinephric adipose tissue; (**d**) Low power view showing the primary PRCC confined within the capsule and perinephric tissue with biopsy site changes with hemorrhage and foreign material deposition (inset); (**e**) perinephric tissue with foreign body reaction (dashed arrow); (**f**) High power view highlighting nests of tumor cells (arrow) seeded in the perinephric adipose tissue

Needle tract tumor seeding within the perinephric adipose tissue was identified in 6 out of 98 (6 %) renal cell carcinoma cases. This was exclusively observed in PRCC (6/28, 21 %), with type 1 features, unifocal, small-sized (≤ 4 cm), and confined to the kidney (Table 2). Histology of the representative cases with tumor seeding along the biopsy needle tract is shown in Fig. 1. In contrast, none of the other tumors (63 CCRCC, 3 CCPRCC, and 4 ChRCC) showed tumor seeding along the biopsy needle tract. In four of the six cases, the presence of tumor cells within the perinephric adipose tissue associated with biopsy needle tract were documented in the pathology reports. Three of these four cases, otherwise pT1a, were upstaged to pT3a due to needle tract tumor seeding. The majority (5/6) of the tumors showed low nucleolar grade (grade 1 or 2). Post-operative follow-up period of the six cases with tumor seeding ranged from 1 month to 52 months with a median of 10.5 months. One patient died of complications from stroke one month following nephrectomy, and one patient was lost for follow up 13 months post nephrectomy. Post-operative follow-up period of the comparable 9 pT1a low grade PRCC cases without tumor seeding ranged from 7 month to 130 months with a median of 36 months; one patient was lost to follow up. No recurrence or metastasis were identified in any of the pT1a PRCC cases, with or without tumor seeding. With regard to the pT3a PRCC cases, one out of the 5 patients developed metastatic lesions in multiple retroperitoneal lymph nodes at 7 months after radical nephrectomy. However, the primary PRCC in this patient had type 2 features, was of high nucleolar grade, and exhibited lymphovascular invasion and lymph node involvement at the time of nephrectomy. The other four patients (one with type 2 features and high nucleolar grade; one with type 1 features and high nucleolar grade; the other two with type 1 features and low nucleolar grade) showed no evidence of local recurrence or metastases during the follow-up period ranging from 24 to 51 months.

**Table 2:** Summary of clinicopathologic features of cases with tumor seeding along the biopsy needle tract

Case	Age (years)	Sex	RCC subtype	Surgical procedure	Tumor stage^a^	Tumor size (cm)	ISUP nucleolar grade	Complete sampling	Follow up
1	59	F	PRCC, type 1	Partial nephrectomy	pT1a	2.3	1	No	1 month, died of stroke
2	55	M	PRCC, type 1	Partial nephrectomy	pT1a	2.1	2	No	11 months, no recurrence
3	70	F	PRCC, type 1	Partial nephrectomy	pT1a	1.7	1	Yes	52 months, no recurrence
4	48	M	PRCC, type 1	Partial nephrectomy	pT1a	1.5	1	No	6 months, no recurrence
5	62	M	PRCC, type 1	Partial nephrectomy	pT1a	1.2	2	Yes	10 months, no recurrence
6	46	M	PRCC, type 1	Partial nephrectomy	pT1a	3.8	3	No	13 months, no recurrence; lost to follow up

^a^ Based on the 8th edition American Joint Committee on Cancer (AJCC) pTNM staging system; biopsy tract seeding not taken into consideration

Abbreviations: RCC, renal cell carcinoma; PRCC, papillary renal cell carcinoma

### Potential impact of specimen sampling and biopsy techniques on biopsy site identification and tumor seeding

To further evaluate whether the observed different rates in needle tract tumor seeding among different histological types of RCC were confounded by the extent of specimen sampling, we evaluated the identification of biopsy site with regard to different approaches of specimen sampling and surgery procedure (Table 3). Interestingly, the biopsy site was identified in 9 of the 28 PRCC cases, and tumor seeding along biopsy needle tract was seen in six cases. In contrast, biopsy site was present in 2 CCRCC cases; neither of the cases showed tumor seeding. Due to small sample sizes, no statistical analysis on the correlation of biopsy site identification and tumor seeding was performed.

**Table 3:** Summary of biopsy site and tumor seeding

	Papillary renal cell carcinoma (PRCC)			Clear cell renal cell carcinoma (CCRCC)			Clear cell papillary renal cell carcinoma (CCPRCC)			Chromophobe renal cell carcinoma (ChRCC)		
	n (%)	Biopsy site, n (%)	Tumor seeding, n (%)	n (%)	Biopsy site, n (%)	Tumor seeding, n (%)	n (%)	Biopsy site, n (%)	Tumor seeding, n (%)	n (%)	Biopsy site, n (%)	Tumor seeding, n (%)
**Partial nephrectomy**	19 (68 %)	9 (32 %)	6 (21 %)	43 (68 %)	0 (0 %)	0 (0 %)	1 (33 %)	0 (0 %)	0 (0 %)	4 (100 %)	0 (0 %)	0 (0 %)
**Radical nephrectomy**	9 (32 %)	0 (0 %)	0 (0 %)	20 (32 %)	2 (3 %)	0 (0 %)	2 (67 %)	0 (0 %)	0 (0 %)	0 (0 %)	0 (0 %)	0 (0 %)
**Entire sampling**^**a**^	7 (25 %)	4 (14 %)	2 (7 %)	9 (14 %)	0 (0 %)	0 (0 %)	1 (17 %)	0 (0 %)	0 (0 %)	0 (0 %)	0 (0 %)	0 (0 %)
**Incomplete sampling**^**b**^	21 (75 %)	5 (18 %)	4 (14 %)	54 (85 %)	2 (3 %)	0 (0 %)	2 (67 %)	0 (0 %)	0 (0 %)	4 (100 %)	0 (0 %)	0 (0 %)

^a^ refers to the specimens entirely submitted for microscopic examination

^b^ refers to the specimens with representative sections submitted for microscopic examination

Of all the 98 cases, 17 tumors were entirely submitted for microscopic evaluation, while the other 81 tumors were incompletely sampled. The biopsy sites were identified in 23 % of cases with complete tumor sampling and 9 % of cases with incomplete tumor sampling. Although the differences on the frequencies of identifying biopsy site changes were not statistically significant (*p* = 0.08), thorough specimen sampling appeared to lead to a higher chance of identifying these changes. It is evident that the extent of sampling of the perinephric fat would be directly relevant to the likelihood of identifying the biopsy site. However, data on the extent of perinephric fat sampling was not available based on the gross descriptions for most cases. Whether a suspicious biopsy tract site was identified was not mentioned in any of the cases. Regarding the surgical approach, 67 and 31 cases were partial and radical nephrectomies, respectively. Biopsy sites were identified in 13 % of cases with partial nephrectomy and 6 % of cases with radical nephrectomy.

We also evaluated whether there is any effect of renal tumor biopsy techniques on the frequency of tumor seeding in PRCCs, by comparing a few biopsy parameters between pT1a cases with and without tumor seeding (Table 4). The biopsy parameters we looked into are those considered potentially affecting the risk of biopsy tract tumor seeding in the literature, including the biopsy needle size (smaller diameter associated with lower frequency of tumor seeding), use of coaxial sheath (associated with lower chance of tumor seeding), and the number of passes (controversial, but generally speaking, multiple passes associated with higher risk of tumor seeding) [20, 21]. Biopsy procedure information was available in all the 6 cases with tumor seeding and 8 cases without tumor seeding. Among these cases, there are no significant differences in the following parameters, biopsy needle size (*p* = 0.78), application of coaxial sheath technique (*p* = 0.53) and number of passes (*p* = 0.69), between cases with and without tumor seeding.

**Table 4:** Renal tumor biopsy techniques in pT1a papillary renal cell carcinoma cases with and without tumor seeding

Case	Biopsy needle size (gauge)	Coaxial sheath technique	Number of passes	Tumor seeding
1	18	No	2	Yes
2	18	Yes	4	Yes
3	18	Yes	3	Yes
4	18	Yes	5	Yes
5	18	Yes	6	Yes
6	18	No	2	Yes
7	18	Yes	3	No
8	18	Yes	3	No
9	20	Yes	6	No
10	18	No	4	No
11	20	No	6	No
12	18	No	3	No
13	18	No	3	No
14	20	Yes	4	No

## Discussion

Our present study is one of the largest case series from a single institution to date evaluating the incidence of biopsy needle tract tumor seeding confirmed by histological examination. It is also the first study investigating the differential frequencies of tumor seeding among specific histologic subtypes of RCC. In our cohort, tumor seeding within the perinephric adipose tissue along the biopsy needle tract was observed in 6 % (6/98) of all RCC resection cases, but exclusively among patients with PRCC (6/28, 21 %). The previously reported overall tumor seeding rate ranges from 0.01 % [9, 10] to 1.2 % [16] in the literature. The lower tumor seeding rates reported in prior studies in the 1990 s were estimated based on responses to questionnaires at multiple institutions [9, 10]. Although the total number of biopsies in the prior studies was large (more than 10,000 biopsies of abdominal masses including renal masses), no standardized protocols on detection of tumor seeding were described, likely resulting in underestimation of the frequency of tumor seeding. Microscopically, we observed clear histologic evidence of biopsy tract changes with intermingled tumor cell clusters, discontinuous from the main tumor, in all the six cases that we interpreted as needle tract tumor seeding. However, there is a lack of specific histologic criteria in interpretating biopsy according to a recent multi-institutional survey and interobserver variability exists [22], which could potentially contribute to the variable frequencies of needle tract tumor seeding reported.

To date, there are a total of 25 reported cases on tumor seeding along the percutaneous renal mass biopsy tract, from several case reports and one case series [8, 11–16]. Of these, PRCC (15 cases) was the most commonly encountered pathologic subtype. Other histologic subtypes included 3 CCRCC, 4 renal cell carcinomas (subtypes not specified), 1 oncocytoma, 1 urothelial carcinoma of the kidney and 1 “angiomyoliposarcoma”. Although this phenomenon was observed in several renal tumors, a predilection for tumor seeding was identified in PRCC compared to other types.

We did not observe biopsy needle tract tumor seeding in CCRCC in our case series, while rare cases of tumor seeding in CCRCC were published previously [8, 12]. Biopsy tract seeding was not observed in CCPRCC and ChRCC either, but the sample sizes for these two subtypes were small. We evaluated several factors possibly affecting detection of tumor seeding in the resection specimens. Although the tumors along with perinephric tissue were sampled per CAP (College of American Pathologists) protocols, not all were entirely sampled (especially the larger-sized tumors). Our data suggests that gross sampling might influence microscopic identification of biopsy site changes. Moreover, careful gross examination of the nephrectomy specimen for scarring, fat necrosis, hemorrhage and fibrosis in the perinephric fat or hemorrhagic foci in the capsular surface, and more diligent sampling of such areas, if present, might help identify sites of needle tract, thus allowing more efficient evaluation for potential tumor seeding [19]. In our series, no macroscopic descriptions of suspected biopsy tract were mentioned upon retrospective review of all the cases, suggesting no targeted samplings for biopsy tract. However, the drastic difference in the tumor seeding rate between CCRCC and PRCC is not readily explained by tumor sampling alone. A few theories were proposed in the literature to explain the higher frequencies of needle tract tumor seeding in PRCC. Some studies observed that PRCC tend to exhibit incomplete or absent peritumoral pseudo-capsule more frequently than CCRCC, facilitating tumor invasion into the perinephric fat [23]. Other hypotheses for higher rate of needle tract tumor seeding in PRCC include the friable nature of the tumor facilitating tumor cell adherence to the needle, higher frequency of exophytic growth allowing tumor seeding more often in the extrarenal space, as well as possible higher chance of tumor cell survival when explanted into the needle tract [17]. Nevertheless, the exact reasons for the differences in the frequencies of biopsy needle tract tumor seeding among various pathologic types of renal tumors need further investigation.

Pathologic staging of renal cell carcinoma is one of the essential prognostic factors and guides patient management, especially surveillance following surgery. Localized pT1a or pT1b renal cell carcinomas are considered as low-risk disease, with recurrence risk of 1–8 %. For these patients, abdominal imaging is recommended annually for 3 years. In contrast, patients with localized T2 and higher disease are considered as having moderate- to high-risk of recurrence (30–78 %). More intensive surveillance protocol is warranted with abdominal imaging (CT or MRI) recommended at 3-6-month interval for the first 3 years, then annually to the fifth year [24].

To date, there is no evidence-based standard protocol among pathologists on whether upstaging is justified solely based on the finding of perinephric tumor seeding along biopsy needle tract. In prior reports, one case of PRCC with tumor foci involving perinephric fat was initially staged as pT3a [14]. However, following confirmation that the tumor foci represented tumor seeding of prior biopsy tract within perinephric fat, the final stage was revised from pT3a to pT1a, indicating that the authors did not consider perinephric tumor seeding along biopsy tract as true cancer invasion [14]. In contrast, in a seven-case series with tumor seeding along the biopsy needle tract involving perinephric fat, six of seven tumors (PRCC and CCRCC) were upstaged to pT3a solely due to biopsy tract seeding, which would have been otherwise staged as pT1a [16]. Understanding the biological behavior of tumor cells spread along the biopsy tract is fundamental to ascertain appropriate cancer staging. It is questionable whether passive displacement of potentially indolent tumor cells to a location that would theoretically necessitate upstaging is equivalent to a genetically aggressive counterpart that actively invades the perirenal tissue. For example, a review on tumor seeding following breast needle biopsy found that the incidence of detecting tumor seeding declines as the interval between biopsy and surgery lengthens, suggesting reduced viability of the seeded tumor cells [25]. On the other hand, it could be argued that an increased access to lymphatic structures and blood vessels in the perirenal tissue by tumor seeding may play a more important role. Thus far, basic mechanistic studies and long-term clinical follow-up data are sparse. It is unclear whether and how the pathologic features of the original tumor and microenvironment of tumor seeding site would affect tumor regrowth. It may also be technically challenging to determine the casual relationship between recurrence/metastasis at a later time and prior tumor seeding phenomenon. The follow-up data based on our series of PRCC seem to show a low risk of recurrence between patients with low grade pT1a disease with and without tumor seeding in the perinephric adipose tissue. Studies with a larger patient cohort and longer follow-up are needed for a more definitive prognostic assessment. Despite these uncertainties, it is documented that two (CCRCC and RCC not specified) of the 25 previously published cases with perinephric biopsy tract tumor seeding showed local cancer recurrence associated with prior biopsy site. Moreover, seven cases (RCC not specified, CCRCC, PRCC, and oncocytoma) exhibited extrarenal subcutaneous or retroperitoneal tumor nodules histologically consistent with the original renal tumors. Therefore, thorough and diligent grossing and microscopic examination for biopsy site changes and signs of tumor seeding is recommended, especially in small-sized tumors, the management and/or follow-up of which may differ significantly based on whether tumor is confined to the kidney or not. Effective communication between pathologists and clinicians and precise documentation of the tumor seeding is essential to facilitate appropriate follow up and patient management.

There are several limitations to our study. First, due to the retrospective nature of the study, we were not able to ascertain whether evidence of needle tract changes was diligently looked for and adequately sampled during grossing. Second, the lengths of follow-up for the six cases with biopsy tract tumor seeding were relatively short, limiting the long-term evaluation of prognosis. Third, the number of cases for CCPRCC and ChRCC were relatively small, limiting the study of tumor seeding in these two subtypes.

Tumor seeding along the biopsy needle tract in patients with RCC warrants increased attention due to its higher frequency than previously documented and the potential impact on patient management. Future studies on a larger scale and longer follow up to evaluate the association between needle tract tumor seeding and prognosis are warranted.

## Data Availability

All data generated or analyzed during this study are included in this published article.
